# Fibrinography: A Multiwavelength Light-Scattering Assay of Fibrin Structure

**DOI:** 10.1097/HS9.0000000000000166

**Published:** 2019-01-24

**Authors:** Carhel Dassi, Landry Seyve, Xabel García, Emmanuelle Bigo, Raphaël Marlu, François Caton, Benoît Polack

**Affiliations:** 1 Laboratoire TIMC-IMAG/TheREx, Université Grenoble Alpes, CNRS UMR 5525, Département d’Hématologie, Institut de Biologie et de Pathologie, Centre Hospitalier Universitaire Grenoble—Alpes, Grenoble, France; 2 Laboratoire Rhéologie et Procédés, Université Grenoble Alpes, CNRS UMR 5520, Grenoble, France

## Abstract

We have previously developed a fibrin structural assay dedicated to purified fibrinogen-thrombin system. Here, we extend the pertinence of this test, called Fibrinography, to tissue factor-triggered plasma coagulation. We show that Fibrinography determines quantitatively the structure of fibrin fibers in plasma with an excellent reproducibility. We compare this assay with the commonly used single wavelength turbidity method, showing that the latter is not a proper structural assay, but determines essentially the fibrinogen content in plasma. In addition, we also show, in model plasmas, that Fibrinography is able to discriminate normal and hypocoagulant plasmas, and even between hypercoagulant plasmas. Therefore, Fibrinography, by measuring the final step of the coagulation cascade, may be used to evaluate patients’ plasma in hypo- or hypercoagulant diseases.

## Introduction

Fibrin formation is the final step of blood clotting and results from the activation of fibrinogen by thrombin generated through the coagulation cascade. Fibrin structures the clot and is responsible for its mechanical properties and resistance to lysis by plasmin.^1,2^ Numerous studies have linked fibrin structure and mechanical properties to pathophysiological situations, as recently reviewed.^3–8^ Indeed, the link between thrombin generation and the mechanical properties of the clot is through the multiscale structure of the fibrin clot. A rather large number of methods have been used to study the mature structure of the fibrin clot at different scales. For instance, fibrin structure has been studied by using “direct” methods such as electron and confocal microscopy,^9^ X-ray or neutron scattering,^10–12^ or using “indirect methods,” such as viscoelastic^13^ and spectral analysis,^13^ clot permeation,^7^ or light scattering.^11,12,14–17^ However, the use of direct methods is restricted to very specialized laboratories especially for X-ray and neutron scattering, while microscopy methods are not well adapted to kinetic measurements and, therefore, not suited to clinical environment. On the other hand, most indirect methods are little adapted to clinical investigations for practical reasons (blood volume, absence of normalization, test duration, etc.) while turbidimetry, which is a form of light scattering, looks most promising given its apparent simplicity.

Since the seminal work of Casassa in 1955,^18^ several groups attempted to deduce quantitatively the radius and mass-to-length ratio of fibrin fibers from wavelength-resolved turbidity data.^11,12,17,19^ Carr and Hermans^17^ further argued that, for sufficiently thin fibers, their mass-to-length ratio could be directly determined from a single wavelength turbidity measurement of a mature clot. This result, which was obtained in purified system (fibrinogen and thrombin), has since been shown to be invalid for plasma.^20^ Despite this unambiguous result, it is still widely believed that the turbidity of a plasma clot is essentially proportional to the thickness of the fibers, and, therefore, used in a large number of studies, as reviewed by Undas and Ariëns.^7^

**Figure 1:**
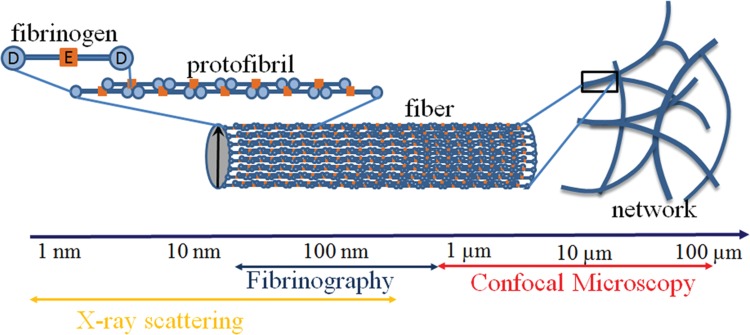
**Multiscale structure of the fibrin clot.** Fibrinography, based upon light scattering, is at the intersection of X-ray scattering and confocal microscopy.

We have previously shown that the limitations of turbidity based methods may be overcome by the use of a multiwavelength approach.^12^ In a purified system it was well suited to explore the noncoagulant effects of heparins on the structure of the fibrin network.^11^ Fibrinography, allowing to measure over time the structure of the forming and mature clot, is the transposition to plasma of this multiwavelength light scattering method that we developed and validated in purified systems.^12^Figure 1 shows that Fibrinography measures the average content of protofibrils inside fibrin fibers.

## Materials and methods

### Materials

Immuno-depleted lyophilized TFPI (tissue factor pathway inhibitor, def-TFPI) and PS (protein S, def-PS) plasmas were obtained from Diagnostica Stago (Asnières, France); their fibrinogen concentrations were 2.2 and 2.4 g/L, respectively. Lyophilized heparinized plasma (calciparin 0.2 UI/mL, Diagnostica Stago) had a final fibrinogen concentration of 2.8 g/L. A frozen normal plasma pool (NP) was obtained from normal donors; its final fibrinogen concentration was 2.5 g/L (Diagnostica Stago). Therefore, there were 3 kinds of plasmas studied: 2 hypercoagulants plasmas (def-TFPI and def-PS), 1 hypocoagulant plasma (heparinized), and 1 normocoagulant plasma (NP). Purified human fibrinogen was from Hyphen Biomed (Neuville-sur-Oise, France).

Plasma fibrinogen concentrations were determined with the Clauss method using Fib5 reagent on a STAR coagulometer (Diagnostica Stago).

### Fibrinography

Initiation of coagulation activation was realized using a method close to the one of Hemker et al.^21^ Briefly, 30 μL of 12 pM of tissue factor (TF) and 24 μM phospholipids (PL) (Thrombinoscope BV, Maastricht, the Netherlands) were mixed with 120 μL of plasma and clotting was triggered upon addition of 30 μL CaCl_2_. Final concentrations were 2 pM TF, 4 μM PL, and 16.7 mM CaCl_2_. Light scattered by the forming clot was measured from 500 to 820 nm (2.5 nm resolution), every 6 seconds during 30 minutes at 37°C (±0.5°C) in a microplate reader (SPECTROstar Omega, BMG Labtech, Ortenberg, Germany). This microplate reader, using a xenon flash light and a CCD sensor, allows the acquisition of a full spectrum at once.

From those spectra, Fibrinography determines the temporal evolution of the number of protofibrils inside fibrin fibers as well as the radius of those fibers during plasma clot formation, as shown in Fig. 1. Briefly each wavelength-dependent optical density spectrum is related to the structure of the fibrin fibers according to Eq. (1):^12^ 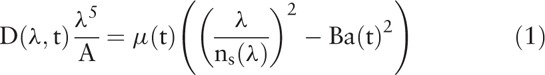


with
 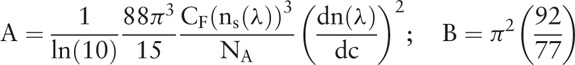


where D is the optical density, t the time, λ the wavelength, μ the mass-to-length ratio of the fibrin fibers; a the radius of the fibrin fibers and C_F_ the fibrinogen concentration, n_s_(λ) the refractive index of water, dn/dc the refractive index increment, and N_A_ the Avogadro number.


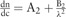
 with A_2_ = 0.1863 and B_2_ = 2.15 × 10^−3^ cm^3 ^μm^2^/g


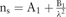
 with A_1_ = 1.327 and B_1_ = 3.06 × 10^−3^ cm^3 ^μm^2^/g^22^

Note that optical density and turbidity are related by τ = D ln(10) where D is the optical density since turbidity (τ) = −ln(I_t_/I_o_) and absorbance or optical density (OD) = −Log(I_t_/I_o_), where I_t_ stands for the transmitted intensity and I_o_ for the incident intensity. Since Eq. (1) shows that τ(t,λ) λ^5^/A is a linear function of (λ/n_s_)^2^, the mass-to-length ratio μ and the radius *a* of the fibers are determined at each time step by a linear fit. The number of protofibrils is determined at each time step from the mass-to-length ratio: Np = μ/μ_0_ where μ_0_ = 1.44 × 10^11^ Da/cm is the mass-to-length ratio of a protofibril.^12^ The length of the optical path was determined by an absorbance measurement and OD was adjusted to a 1 cm path.

### Thrombin generation

Thrombin generation was measured using the Calibrated Automated Thrombogram (CAT) initiated by automatically dispensing fluorogenic substrate (Z-Gly-Gly-Arg-AMC) in CaCl_2_ (416 μM and 16.7 mM, final, respectively), calibrated against wells containing α_2_-macroglobulin/thrombin complex and plasma, and analyzed with Thrombinoscope software (Thrombinoscope BV), according to Hemker's method.^21^ Final TF and PL concentrations were 2 pM and 4 μM, respectively, as in Fibrinography.

### Confocal microscopy

Fluorescent fibrinogen (Human Fibrinogen Alexa Fluor 488 conjugate, Thermo Fisher Scientific, Villebon sur Yvette, France) was added to plasma at a 1/10 ratio of fibrinogen plasma concentration. Then 200 μL of this mixture was incubated with 50 μL of TF and PL (respectively 2 pM and 24 μM final concentrations), and clotting was triggered by adding 50 μL of CaCl_2_ (16.7 mM, final concentration). One hundred confocal stack images over a depth of 50 μm were taken after 30 minutes of fibrin network formation at 37°C. Images at different depth were compared to ensure that the network was homogeneous. When appropriate, pore size was determined using the method proposed by Munster and Fabry.^23^

## Results

### Fibrinography

**Figure 2:**
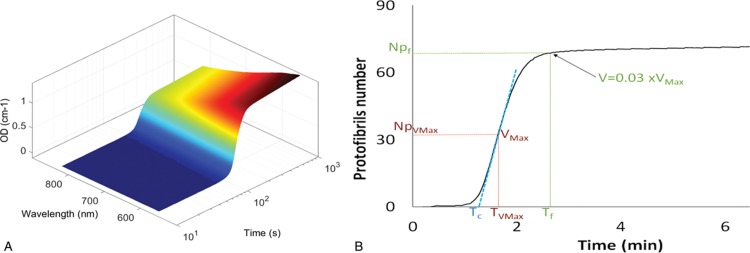
**Temporal evolution of optical density spectra during clot formation and calculated Fibrinography.** Plasma was coagulated at 37°C in the presence of 2 pM TF, 4 μM PL, and 16.7 mM CaCl_2_. Optical density spectra from 500 to 820 nm was recorded over time and plotted (panel A). Fibrin structure was then calculated according to Eq. (1) as described in the Materials and Methods Section. Fibrinography (panel B) shows the temporal evolution of the number of protofibrils for the same experiment as in the left panel. Characteristic times are: the clotting time (T_c_, extrapolation to the abscissa of the tangent to the maximum velocity), the time of maximum velocity (T_Vmax_, corresponding to the inflexion point of the curve), and fibers growth time (T_f_) defined as the point where the maximum velocity slows to 3% of its maximum.

The typical evolution of optical density spectra during a clotting experiment is shown in Fig. 2A. From those data, the number of protofibrils inside the fibers as well as the radius of the fibers are extracted using Eq. (1) as described in the Materials and Methods Section. Therefore, Fibrinography reports the temporal evolution of the number of protofibrils as plotted in Fig. 2B. We define several times corresponding to characteristic features of Fibrinography, as shown in Fig. 2B:

- The time of maximum reaction velocity (inflexion point), T_Vmax_
- The clotting time, T_c_: extrapolation on the abscissa of the maximum velocity tangent
- The fibers formation time (reaction velocity down to 3% of max velocity), T_f_
- The protofibrils numbers at those characteristic times: NP_Vmax_, NP_c_, and NP_f_ that is the one represented throughout this work

### Reproducibility

**Figure 3:**
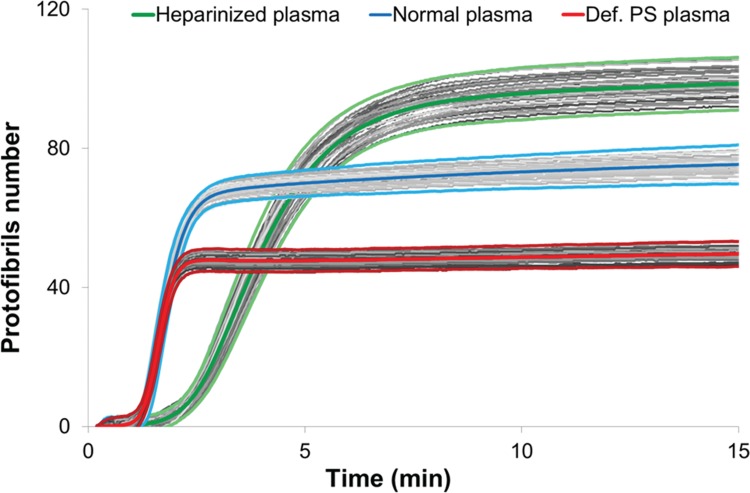
**Fibrinography reproducibility.** Fibrinography was performed as described in material and methods for our normal plasma pool (blue curves, fibrinogen = 2.5 g/L), heparinized plasma (green curves, fibrinogen = 2.8 g/L), and def-PS plasma (red curves, fibrinogen = 2.4 g/L). For each plasma, experiment was repeated 40 times on different days. All the curves are represented on the graph with the individual curves in grey centered by the mean curves (thick colored curves). All the individual curves are contained in the ±2 standard deviations curves (light colored curves).

We then evaluated the reproducibility of Fibrinography by replicating the measure 40 times for hyper-, hypo-, and normocoagulant plasmas, on several days. Figure 3 shows the reproducibility of the assay where coefficients of variation (CV) for the normal and PS depleted plasmas were lower than 5%, while culminating to 8% for the heparinized one (Table 1). In addition, all replicates were within 2 standard deviations. As expected, the clotting time for the heparinized plasma was longer than for the other 2.

**Table 1:**
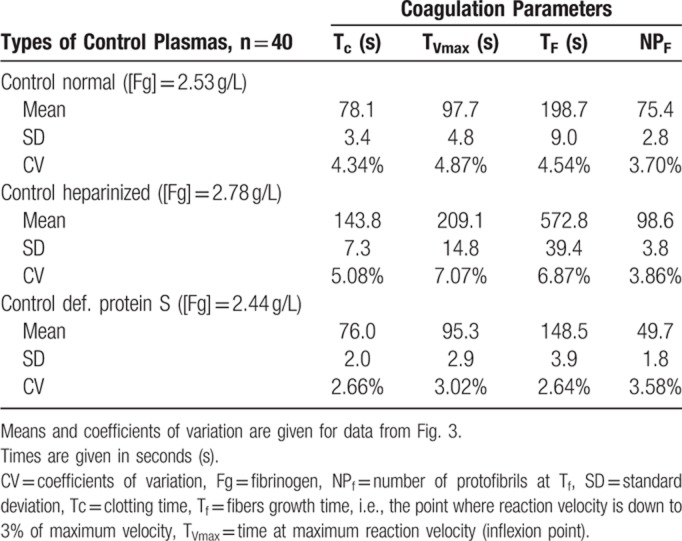
Coagulation Times and Number of Protofibrils.

### Effect of the coagulation cascade on Fibrinography

In order to analyze the effect of thrombin on Fibrinography, we next compared Fibrinography to thrombin generation (CAT) for normal, hyper- and hypocoagulant plasmas. To that end, we performed both assays under the same conditions in terms of TF, PL, and Ca^2+^ concentrations.

**Figure 4:**
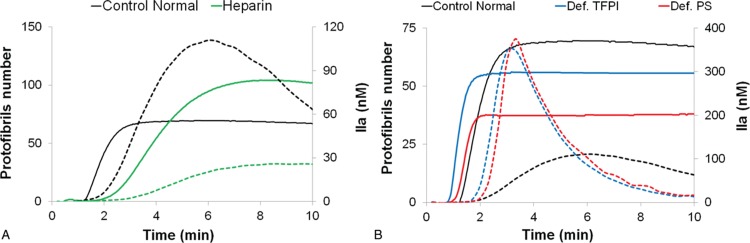
**Effect of coagulation on Fibrinography and thrombin generation assays.** Fibrinography and CAT were performed using the same conditions, as described in the Materials and Methods Section, with 2 pM TF, 4 μM PL, and 16.7 mM CaCl_2_. (A) Hypocoagulant heparinized plasmas, green curves. (B) Hypercoagulant plasmas depleted either in TFPI, blue curves, or in PS, red curves. Normal plasma, in black, is present on both panels. Continuous curves stand for Fibrinography and dashed curves for CAT assays.

Figure 4A shows both Fibrinography (continuous lines) and thrombin generation (dashed lines) for normal and hypocoagulant (heparinized) plasmas. Within 10 minutes, there is a clear discrimination between the 2 plasmas. Heparinized plasma coagulates slowly, generates less thrombin, and exhibits a higher number of protofibrils than the normal one. Indeed, as shown in Table 1, the heparinized plasma has 35% more protofibrils per fiber than the normal one. We then compared 2 hypercoagulant plasmas (def-TFPI and def-PS) to the normal one. The CAT assay (dashed lines) shows a higher thrombin generation for the 2 hypercoagulant plasmas. However, their thrombin generation curves are rather similar. Conversely, Fibrinography (continuous lines) shows a clear difference in protofibril numbers of roughly 20% between the 2 hypercoagulant plasmas (Fig. 4B).

### Effect of fibrinogen concentration

**Figure 5:**
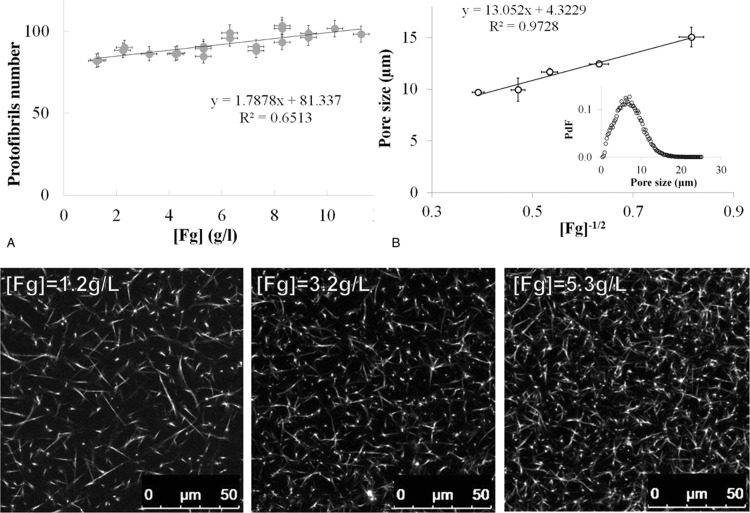
**Effect of fibrinogen concentration of Fibrinography.** The 50% diluted normal plasma pool was spiked with purified fibrinogen in order to generate fibrinogen concentrations ranging from 1.2 (diluted plasma) to 11.3 g/L. Plasma was coagulated at 37°C as described in the Materials and Methods Section with 2 pM TF, 4 μM PL, and 16.7 mM CaCl_2_. (A) Final protofibrils number as a function of fibrinogen concentration (mean ± SD). Bottom panel: Confocal microscopy images obtained for selected concentrations of fibrinogen. (B) Confocal microscopy images were analyzed according to Munster and Fabry^23^ to determine the pore sizes inside the fibrin clots (mean ± SD). A typical histogram of pore size is represented in the inset. PdF = pore size probability distribution function.

We then evaluated the effect of fibrinogen concentration on Fibrinography. To that aim, we determined the number of protofibrils from clots obtained in plasma with concentrations of fibrinogen ranging from 1.2 to 11.3 g/L. This range of fibrinogen concentration was obtained upon the addition of various concentrations of purified human fibrinogen to a 50% dilution of our normal plasma pool. The plasma dilution was held constant and thrombin generation was determined showing a gradual increasing in thrombin generation (data not shown) as previously described.^24^Figure 5A shows that the number of protofibrils obtained by Fibrinography is almost independent of fibrinogen concentration with only a 20% increase between 1 and 12 g/L. In parallel, we obtained confocal microscopy images stacks of the same plasma + fibrinogen system at several concentrations of fibrinogen (Fig. 5, bottom panel). As can be seen, fibers density increases in parallel with the concentration of fibrinogen. Furthermore, the pore size measurements follow a 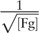 law (Fig. 5B).^25^

### Comparison with single wavelength turbidimetry

**Figure 6:**
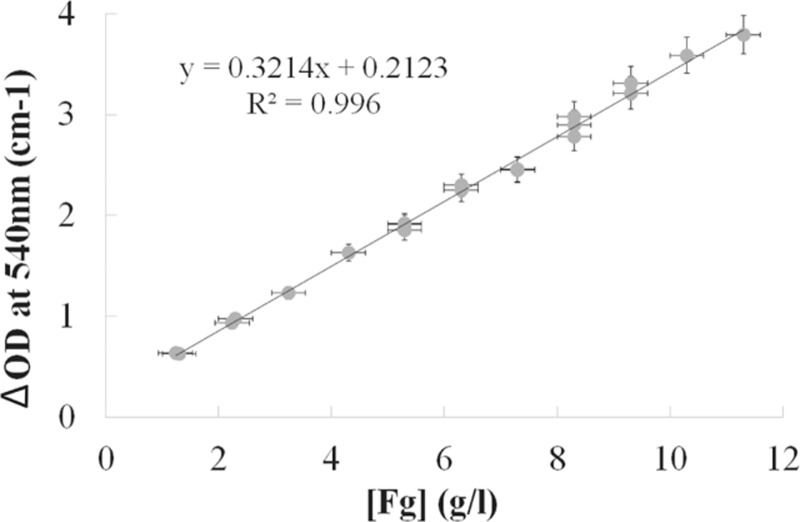
**Effect of fibrinogen concentration on the single wavelength optical density of the clot.** The 50% diluted normal plasma pool was spiked with purified fibrinogen in order to generate fibrinogen concentrations ranging from 1.2 (diluted plasma) to 11.3 g/L. Plasma was coagulated at 37°C as described in the Materials and Methods Section with 2 pM TF, 4 μM PL, and 16.7 mM CaCl_2_. Final optical density at 540 nm was extracted from the spectra and plotted over fibrinogen concentration expressed as mean ± SD.

Up to date, fibrin's structure has usually been qualitatively estimated by measuring the total turbidity variation during clotting (see review by Undas and Ariëns^7^) at a single wavelength. In order to compare the results of Fibrinography with this estimation, we used the above experiments with fibrinogen addition to normal plasma. Since we measured the complete 500 to 820 nm optical density wavelength spectra in those experiments, we extracted from those spectra the optical density at the commonly used wavelength of 540 nm (OD_540_). Figure 6 shows that the total optical density variation is directly proportional to the fibrinogen concentration with a perfect correlation (R^2^ = 0.996). This result is identical whatever the chosen wavelength is. It demonstrates that measuring the turbidity at a single wavelength determines essentially the concentration of fibrinogen.

**Figure 7:**
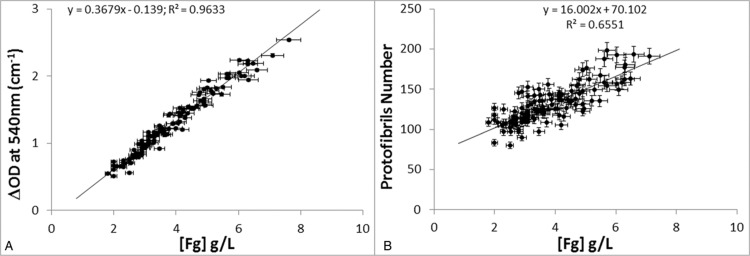
**Effect of fibrinogen concentration on Fibrinography in patients’ plasmas.** Residual plasma samples from 141 patients were coagulated as described in the Materials and Methods Section, at 37°C with 2 pM TF, 4 μM PL, and 16.7 mM CaCl_2_. Optical density spectra were recorded over time and either Fibrinography was determined (panel A) or optical density at 540 nM was extracted (panel B) and plotted over fibrinogen concentration determined by the Clauss method. Results are presented as mean ± SD.

In addition, we performed Fibrinography on residual plasma samples from 141 patients obtained from the hematology department. Coagulation was initiated as described in the Materials and Methods Section and the OD wavelength spectra were recorded from which both the number of protofibrils was determined and the turbidity at 540 nm (OD_540_) was extracted and compared to fibrinogen levels. Figure 7 shows that the behaviors of single wavelength turbidimetry and of Fibrinography differ very strongly. The optical density at 540 nm is again proportional to the fibrinogen concentration with a fine correlation (Fig. 7A, R^2^ = 0.96). Conversely, the number of protofibrils measured by Fibrinography shows a weak correlation with fibrinogen concentration (Fig. 7B, R^2^ = 0.66), and a much larger dispersion of data.

## Discussion

We have previously shown that the method of Carr and Hermans,^17^ despite its large use in the literature, is not appropriate to measure the protofibril content of fibrin fibers from clots obtained in purified systems.^12^ We modified this method to obtain coherent results^12^ and show here that this improved method is also pertinent in plasma activated by calcium chloride in the presence of picomolar concentrations of tissue factor and procoagulant phospholipids. Moreover, we were also able to show that it was possible to use it in a more physiological setting than plasma clotted by thrombin (e.g.,^20^), that is, in a way similar to the largely used thrombin generation test proposed by Hemker et al.^21^ In addition, and similarly to TGT, we were also able to monitor over time the formation of the fibrin clot, as shown here in term of protofibril number (Fig. 2B), and with a good reproducibility (Fig. 3).

In our previous experiments in purified systems, we have confirmed the results of our optical method using a small-angle X-ray scattering approach.^12^ However, small-angle X-ray scattering is impracticable in plasma since it requires a measurement of the background scattering, that is, without the polymerizing substance. While this can easily be done in purified system since fibrinogen can be added after recording the large background scattering of the buffer, doing it in plasma is more complex. Indeed, it requires removing fibrinogen from plasma to obtain the background while maintaining intact the rest of the protein content in plasma. Therefore, to overcome this problem we took advantage of the fact that, since the total mass of fibrin is conserved, the nanoscale structure, that is, the number of protofibrils in fibrin fibers, is tightly linked to the micron-scale structure, that is, the size of the pore between the fibrin fibers determined through our confocal microscopy observations. For instance, if the nanoscale structure is constant, the size of the pores of the fibrin network must be proportional to the inverse of the square root of the fibrinogen concentration as previously described.^25,26^ Being what we observed actually in Fig. 5B, it highlights the objectivity of Fibrinography. More fundamentally, this result agrees with and extends those obtained in purified systems using the same methods^25,26^ as well as small-angle neutron scattering experiments which demonstrated directly that the number of protofibrils within the fibers slightly increases with fibrinogen concentration, with exactly the same variation than observed here.^10^

We also compared Fibrinography to the widely used turbidity measurement performed at a single wavelength (e.g.,^7^ and references herein). Our results demonstrate that measuring the optical density at a single wavelength mainly determines the fibrinogen concentration in the sample, as expected from the similarity of this assay with the prothrombin time-derived fibrinogen determination,^27–29^ rather than the structure of the clot. Therefore, maximum turbidity in plasma clots is not a determinant of fibrin's structure, but is rather a measure of the fibrinogen concentration, even in the case of patients’ plasma as shown recently by Jacquemin et al.^29^ Besides, when comparing data from patients (Fig. 7) with the fibrinogen addition experiment (Fig. 5), the data points observed from patients are considerably more dispersed, and the linear coefficient is almost ten times larger (16.0 vs 1.8), indicating that fibrin's structure depends slightly on fibrinogen concentration but strongly on the coagulant potential of the patient's plasma.

This dependence upon coagulation was also highlighted by our models of hypo-, normo-, or hypercoagulant plasmas. Actually, Fibrinography discriminated hypo-, normo-, or hypercoagulant plasmas, but, moreover, was able to show differences between 2 hypercoagulant plasmas. Indeed, the def-PS plasma was “more” hypercoagulant than the def-TFPI as during the immune-depletion of PS the TFPI linked to it was also eliminated, since the 2 proteins are associated in plasma.^30,31^

## Conclusion

In conclusion, we have shown that Fibrinography determines the proper nanostructural parameters of clots obtained in plasma coagulated by tissue factor, as demonstrated by comparison with confocal microscopy and as previously shown in purified system by comparison with small angle X-ray scattering.^12^ Conversely, we also demonstrated that single wavelength turbidimetry is not a structural assay. Indeed, the maximum optical density of the clot reflects essentially its fibrinogen concentration while being only marginally related to clot structure. We also show that Fibrinography was able to discriminate hypercoagulant plasmas. Since Fibrinography is easy to perform on a spectrophotometer microplate reader it may be implemented in clinical laboratories in complement of thrombin generation test. Further evaluations in clinical settings are needed to confirm that Fibrinography may become the global hemostasis test that Lipets and Ataullakhanov^32^ and Lancé^33^ hoped for.

## Acknowledgment

We acknowledge fruitful discussions with G. Contant and O. Mathieu.
